# A New Dual-Promoter System for Cardiomyocyte-Specific Conditional Induction of Apoptosis

**DOI:** 10.1155/2013/845816

**Published:** 2013-01-31

**Authors:** Silvia Agostini, Vincenzo Lionetti, Marco Matteucci, Flavia Chiuppesi, Mauro Giacca, Mauro Pistello, Fabio A. Recchia

**Affiliations:** ^1^Gruppo Intini-SMA Laboratory of Experimental Cardiology, Institute of Life Sciences, Scuola Superiore Sant'Anna, 56127 Pisa, Italy; ^2^Retrovirus Centre and Virology Section, Department of Translational Research, University of Pisa, 56127 Pisa, Italy; ^3^Molecular Medicine Laboratory, International Centre for Genetic Engineering and Biotechnology, Padriciano, 34149 Trieste, Italy; ^4^Department of Physiology, Temple University School of Medicine, Philadelphia, PA 19140, USA

## Abstract

Apoptosis is a key determinant of major pathological processes, including chronic cardiac failure. We developed and tested *in vitro* a novel system to induce cardiomyocyte-specific apoptosis by virus-mediated delivery of a conditional transgene. The entire system was packaged in a lentiviral vector. We used the cardiomyocyte-specific Na^+^-Ca^2+^ exchange promoter to control the transcription of the reverse tetracycline transactivator, while the transgene expression was driven by the tetracycline-responsive element. The proapoptotic transgene of choice was the short isoform of the apoptosis-inducing factor, known to quickly induce the caspase-independent apoptosis when overexpressed in cells. Transduction of cardiomyocyte cells with this vector caused a tetracycline-regulated induction of apoptosis, which was not observed in noncardiac cells. Therefore, our system proved a valuable molecular tool for inducing controlled apoptosis in selected cells. Furthermore, the vector we developed is suitable for “lentivirus transgenesis,” an interesting strategy recently proposed for the genetic manipulation of animals other than mice, including large mammals.

## 1. Introduction

 Apoptosis is a key process involved in the pathogenesis of major diseases affecting the immune, nervous, gastroenteric, and cardiovascular system [1]. For instance, studies in humans and in dogs have documented the presence of approximately 3000 apoptotic cells per million in tissue harvested from failing hearts [2–4]. This number might seem very small and pathologically insignificant; however, studies in genetically modified mice have shown that a few hundred apoptotic cells per million are sufficient to cause dilated cardiomyopathy [5, 6], a malignant cardiac disease that, in humans, accounts for 50% of the heart transplants [7]. It should also be bear in mind that apoptosis, once triggered, is a rapid process (24–48 h) and consequently any histological evaluation provides a limited “snapshot” of a dynamic and rapid sequence of cell deaths.

Given the importance of apoptosis as a pathogenic mechanism, inducing this particular form of cell death in animal models can provide important insights. Ideally, apoptosis induction should be restricted to selected organs or systems and adequately controlled to avoid fatal massive systemic damage. While such requisites can be met in conditional transgenic mice, genetic manipulations are more challenging in clinically relevant large animal models. A potential strategy in an adult organism is the induction of apoptosis in selected organs by virus-mediated delivery of conditional transgenes. Alternatively, controllable transgenes could be stably incorporated in cells, via viral vectors, during the embryonic life for later activation [8]. However, no vector-carried transgene system, to date, has been developed to induce apoptosis in a controllable fashion. Nevertheless, a possible candidate gene for such a strategy is the apoptosis-inducing factor (AIF).

Two complementary routes leading to programmed cell death have been characterized, namely, the caspase-dependent and caspase-independent pathways, which differ in the events needed to trigger the apoptotic cascade [9]. AIF was the first identified protein involved in caspase-independent cell death [10]. Recent studies have characterized a novel AIF isoform called AIFshort (AIFsh), which comprises 7 exons derived from exon 10 to 16 of AIF. The resulting cytosolic protein corresponds to the C-terminal part of AIF, lacking its N-terminal domain, and is able to trigger caspase-independent cell death upon expression in recipient cells by means of inducing chromatin condensation and large scale (50 kb) DNA fragmentation [11].

Given our specific interest in lentivirus-mediated gene transfer [12, 13] and its cardiovascular applications [14], here, we developed a lentiviral vector for cardiomyocyte-specific, conditional expression of AIFsh. This vector contains two transcriptional units in tandem, one coding for the inducible prokaryotic tetracycline (Tet) activator (rtTA), which was placed under the control of a cardiac-specific promoter, and the second the AIFsh gene regulated by the rtTA-responsive element.

## 2. Materials and Methods 

### 2.1. Cell Lines and Culture

Human renal epithelial (HEK 293T) and HeLa cell lines where cultured in DMEM (Lonza, Basel, Switzerland) supplemented with 10% fetal bovine serum (FBS), glutamine and antibiotics; mouse tumoral cardiac HL-1 cell line (kind gift from Professor W. Claycomb, Department of Biochemistry and Molecular Biology, LSUHSC, New Orleans, LA, USA [16]) was cultured in Claycomb Medium (SAFC, St. Louis, MO, US) supplemented with 10% FBS, antibiotics, glutamine, and norepinephrine (according to Prof. Claycomb recommendations). HL-1 cardiac phenotype and easiness of propagation render these cells a versatile tool as surrogate of cardiomyocytes for *in vitro* studies [16].

### 2.2. Construction of Dual-Promoter Lentiviral Vectors

Cardiac-specific, Tet-regulated lentiviral vectors were developed using an approach similar to the one described by Gascòn and colleagues [15] in neurons. In the Tet-on system, the rtTA transactivator mediates the transcription of a transgene controlled by the TRE inducible promoter. Transcriptional activation is positively regulated by the presence of Tet, which allows the binding of rtTA to the TRE promoter, thereby promoting transgene expression. Therefore, regulated expression with the Tet-on system requires the delivery in the same target cell of both the rtTA transactivator and the transgene.

**Figure 1:**
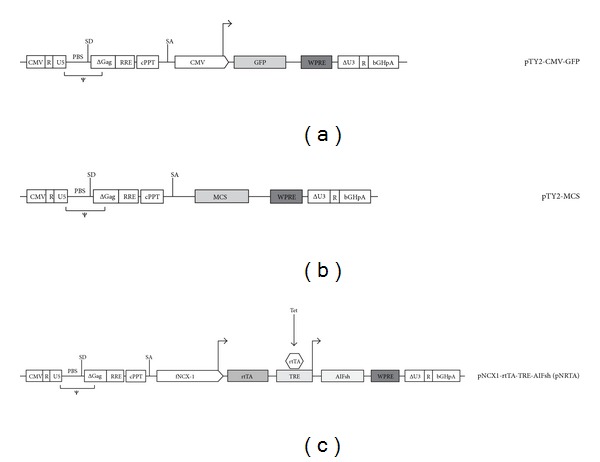
Schematic representation of the parental lentiviral backbone (a), the subsequent cloning intermediate (b), and the final dual-promoter lentiviral vector used in the study (c). A detailed description of backbone components is available elsewhere [15]. The intermediate was derived from the parental clone by removing CMV and GPF; CMV was replaced with the cardiac-specific promoter (NCX1) and rtTA; GFP was replaced by the Tet-responsive element cassette (TRE) and AIFsh. The resulting pTY2-NCX1-rtTA-TRE-AIFsh construct has been named pNRTA for shortness. CMV: cytomegalovirus promoter; GFP, green fluorescent protein; MCS: multicloning site; fNCX1: feline Na^+^-Ca^2+^ exchanger promoter; rtTA-reverse repressor of TRE; TRE, tet-responsive element; Tet: tetracycline; AIFsh: apoptosis-inducing factor-short.

The lentiviral backbone used in this study was derived from pTY2-CMV-GFP, an optimized self-inactivating (SIN), integrase-defective human immunodeficiency virus type-1 (HIV-1) vector obtained by Dr. A. Cara, Istituto Superiore di Sanità, Rome, Italy [17]. For our purposes, the parental pTY2-CMVGFP vector was modified in the multiple cloning site (MCS), which was completely redesigned between the KpnI and SalI restriction sites, and the CMV-GFP cassette that was replaced by an inducible, cardiac cell specific expression system. The inducible expression vector was obtained by inserting the NCX-1 promoter [18], into pTY2-CMV-GFP. This promoter drives the expression of the reverse repressor of the rtTA gene. Another expression cassette downstream contains the TRE sequence placed upstream the hAIFsh coding sequence [11] (Figure 1(c)).

MCS was reshaped by means of two synthetic oligonucleotides, bearing the restriction sites KpnI-AscI-NcoI-EcoRV-BglII-XhoI-XbaI-SacII-NheI-SalI (KpnI and SalI designed as protruding ends upon annealing). The oligonucleotides were phosphorylated with T4 PNK (New England Biolabs, Ipswich, MA, USA), annealed, and then ligated into pTY2-CMV-GFP vector cut with KpnI and SalI enzymes, thus generating the pTY2-CMV-GFP-MCS vector. The CMV-GFP cassette was finally removed by SalI/ClaI digestion followed by Klenow protrusive end blunting and ligation, thus obtaining pTY2-MCS.

AIFsh sequence was amplified from p3xFLAG-AIFsh (a kind gift from Dr. Susin [11]) and cloned in the new MCS between the SacII and NheI sites; TRE element was subcloned from pTRE2 (Clontech Europe, France) by means of digestion with XhoI and SacII and subsequent ligation upstream AIFsh, thus obtaining the pTY2-TRE-AIFsh. Thereafter, rtTA sequence was amplified from pTetON (Clontech Europe, France) and cloned between AscI and EcoRV sites. Finally, the NCX1 promoter sequence was amplified from pNCX1831 plasmid (a gift from Dr. Menick [18]) and cloned into the AscI site upstream of rtTA. The correct orientation and the absence of mutations in all fragments were verified by sequencing. The resulting construct, named pTY2-NCX1-rtTA-TRE-AIFsh (pNRTA), was used for lentiviral vector production.

### 2.3. Lentivirus Production

Recombinant lentiviruses were produced by a three-plasmid transfection procedure as described elsewhere [12, 13, 19]. Briefly, HEK 293T cells were cotransfected with the pNRTA vector or the pTY2-CMV-GFP vector, the packaging vector pΔ8.91, and a vector encoding the G-protein of the vesicular stomatitis virus (VSV-G). 

The virus supernatants were collected 48 hours after transfection, pooled, and filtered through 0.45 *μ*m filters. Viral titers were calculated by measuring HIV capsid p24 titer in the viral supernatants by an enzyme-linked immunosorbentassay (ELISA) (Innogenetics, Gent, Belgium).

### 2.4. Lentivirus-Mediated Transduction

Transduction of cell lines was performed in 12-well plate, 1 mL volume per well, according to Bonci et al. [20]. Lentivirus supernatants were added to 10^5^ target cells (400 ng p24/well, in Opti-MEM 2% FBS) and incubated for 6 hours. Then cells were washed and the medium changed to Claycomb medium 10% FBS. 48 hours after transduction, cells were assayed for inducible apoptosis upon the administration of 1 *μ*g/mL Tet (see below). 

The same protocol was used for HeLa cells with the exception of postinfection medium, which was switched to DMEM 10% FBS.

Efficiency of transduction was evaluated using the same amount of pTY2-CMV-GFP construct and testing the percentage of green cells after 48 hours by flow cytometry. 

### 2.5. Apoptosis Induction and Cell Survival Measuring

Forty-eight hours after transduction, apoptosis was induced upon incubating cells with 1 *μ*g/mL Tet (Sigma Aldrich, St. Louis, MA, USA). Cells were then washed and trypsinized, and live cells were counted by trypan blue exclusion, at 4, 6, 8, and 24 hours of incubation with Tet.

The number of live cells was normalized by the number of live, untransduced, untreated cells at each time point.

### 2.6. DNA Fragmentation Assay (TUNEL), Immunofluorescence

Apoptotic cell death was measured four hours after the addition of Tet by the deoxynucleotidyl transferase-mediated dUTP nick end-labeling (TUNEL) method, using ApoAlert DNA fragmentation assay kit (Clontech Europe, France), and according to the manufacturer's protocol. Images were analyzed using a Zeiss immunofluorescence microscope.

### 2.7. Cell Lysis, Antibodies, and Immunofluorescence

For immunofluorescence analysis, HL-1 cells were grown on coverslips, fixed, and incubated twice for 10 min with phosphate-buffered saline (PBS) 0.1% Triton X-100 to permeabilize plasma membranes; after extensive washing with PBS+ (PBS, 0.1% glycine, and 0.5% BSA), cells were incubated with primary antibody (anti-AIF antibody, 1 : 50) overnight at 4°C. Cells were then washed again in PBS+ and incubated with secondary antibody (donkey anti-goat, Fluorescein isothiocyanate (FITC) conjugated, 1 : 500) for 1 hour at 37°C. Following extensive washing with PBS+, slides were mounted with Vectashield and analyzed. All antibodies were diluted in PBS+.

Goat anti-AIF antibody and donkey anti-goat FITC conjugated were obtained from Santa Cruz Biotechnologies.

### 2.8. Statistical Analysis

The statistical significance was assessed by calculating *P* values from nonparametric two-tailed Student's *t*-test or ANOVA when 3 or more groups were compared. The analyses were conducted using GRAPHPAD Prism 5.00 software.

## 3. Results and Discussion

### 3.1. Dual-Promoter Lentiviral Vectors for Inducible Expression in Cardiomyocytes

Dual-promoter lentiviral vectors for tissue specific expression of selected transgenes have already been described for several highly specialized tissues [21, 22]. Here, we applied and further improved this system to obtain the conditional expression of a transgene in cardiomyocytes. This was achieved by placing AIFsh under control the of the feline Na^+^-Ca^2+^ exchanger (NCX1) cardiac-specific promoter [18] and Tet-inducible activator rtTA. To this purpose, we engineered TY2-CMV-GFP, an HIV-1 vector expressing the green fluorescent protein (GFP) under the control of cytomegalovirus (CMV) promoter. Through the intermediates described in Section 2, the CMV promoter and GFP were replaced by two expression cassettes. The CMV promoter was replaced by the cardiac-specific promoter (NCX1) and rtTA; GFP was replaced by the Tet-responsive element cassette (TRE) and AIFsh. The resulting construct (pTY2-NCX1-rtTA-TRE-AIFsh) has been named pNRTA for shortness (Figure 1).

**Figure 2:**
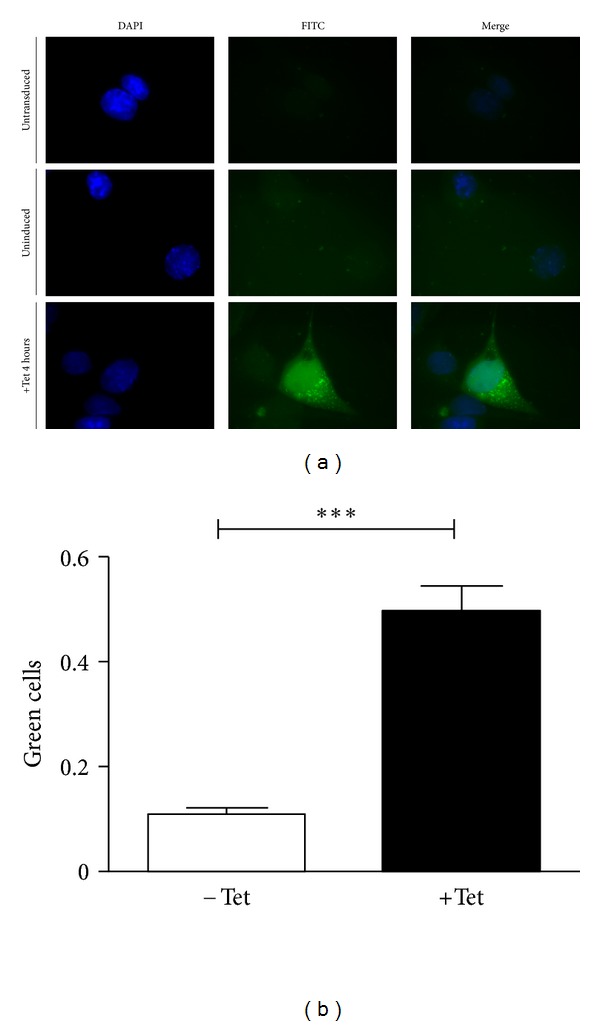
Inducible AIFsh expression in cardiac cells. HL-1 cardiac cells were transduced with the pNRTA vector and assessed for transgene expression 48 hours later. Transgene expression was evaluated before or after induction by exposing cells to 1 *μ*g/mL Tet for 4 hours. (a) Cells were then processed for immunofluorescence with an anti-AIF antibody and an FITC-conjugated secondary antibody. The panel shows sample images of HL-1 cells treated as follows: untransduced (upper), transduced and uninduced (middle), and transduced and induced (lower). DAPI staining, green fluorescence, and a merging of the two channels are shown. (b) Quantitation of immunofluorescent slides of transduced HL-1 cells either uninduced (−Tet) or induced (+Tet). The amount of fluorescent cells is expressed as a fraction of green fluorescent cells over nonfluorescent cells in the field counted.

Cell-specific and Tet-dependent expression of AIFsh was tested by transducing TY2-TRE-AIFsh in mouse cardiac HL-1 cells. The human cervix epithelial adenocarcinoma (HeLa) cells were tested in parallel and used as a negative control. Immunofluorescence analysis with an anti-AIF antibody demonstrated an increase in Tet-dependent expression of AIFsh transgene after Tet induction (1 *μ*g/mL). In contrast, expression was consistently below 10% in nontransduced cells and in transduced, Tet-untreated cells (Figures 2(a) and 2(b)). Similarly, the number of AIFsh-expressing cells in induced versus noninduced cells increased and was a high statistical significance (*P* < 0,001); the number of AIFsh expressing cells was calculated as the ratio between green fluorescent versus nonfluorescent cells in each field counted. The efficiency of transduction was inferred with HL-1 cells transduced with pTY2-CMV-GFP and examined for fluorescence by flow cytometry; the percentage of transduced cells was estimated to be between 60% and 70% in all transduction experiments (data not shown). HeLa cells transduced in parallel showed no transgene expression even after induction (data not shown), thus confirming that the expression was, in fact, cardiomyocyte specific.

### 3.2. Tetracycline-Regulated Transgene Expression

**Figure 3:**
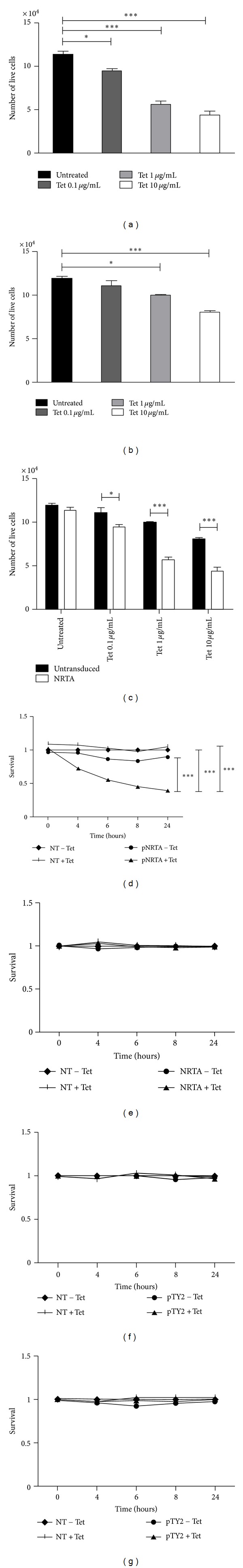
Apoptosis induction and cell survival. 48 hours after transduction with pNRTA vector, apoptosis was induced in HL-1 cells following the exposure to Tet as indicated. Cells were then washed and trypsinized, and live cells were counted by trypan blue exclusion. (a) Number of viable, NRTA-transduced, HL-1 cells after exposure to the indicated amount of Tet for 24 hours. (b) Number of viable, untransduced, HL-1 cells after exposure to the indicated amount of Tet for 24 hours. (c) Number of viable HL-1 cells (transduced and untransduced) after exposition at the indicated amount of Tet for 24 hours. (d) HL-1 cells transduced with pNRTA vector. (e) HeLa cells transduced with pNRTA vector. (f) HL-1 cells transduced with empty pTY2 vector. (g) HeLa cells, transduced with empty pTY2 vector. Cell survival in panels (d) to (g) are expressed as a fraction of live cells over total cells. −Tet: cells unexposed with tetracycline; +Tet: cells exposed to 1 *μ*g/mL tetracycline.

To determine the time-course kinetic of the transgene expression, HL-1 cells were transduced with the inducible vector and cell survival was evaluated after the exposure to Tet. First, the optimal, nontoxic concentration of Tet was estimated by treating cells with the increasing concentrations of the drug and evaluating cell survival after 24 hours. The optimal concentration, that is the best induction with minimal the toxicity versus the similarly treated nontransduced cells was 1 *μ*g/mL (Figures 3(a), 3(b), and 3(c)). This concentration was, therefore, used in the following time-course experiment in which cell survival was measured after 2, 4, 6, 8, and 24 hours of Tet addition and consequent AIFsh induction. Cell counts showed a marked reduction (~50%) in cell survival over time in transduced HL-1 cells treated with Tet, compared to untreated cells (Figure 3(d)). These results indicated that the apoptotic process occurred predominantly between 4 and 24 hours after AIFsh induction, while no further decrease in cell survival was observed at later time points (data not shown). A small percentage of cell death could still be observed in untreated cells and can probably be ascribed to TRE promoter leakage.

To confirm the cell specificity of the observed effect, we performed the same experiments in HeLa cells. Despite good efficiency of transfection (65%), survival of this noncardiac cell line was not affected upon Tet stimulation of gene expression (Figure 3(e)), thus proving that the effect on cell survival is lineage specific and driven by the cardiomyocyte-specific promoter.

To rule out the possibility that the observed reduction of cell survival was either due to Tet treatment or lentiviral transduction, we assessed cell survival in nontransduced HL-1 and HeLa cells, treated or untreated with Tet (Figure 3(e)), and in HL-1 and HeLa cells transduced with the lentiviral backbone lacking the inducible system and the pro-apoptotic transgene (Figure 3(f)). The results clearly showed that the reduction in cell survival was not due to Tet treatment or vector transduction, thus confirming the specificity and inducibility of the system.

### 3.3. Tet Treatment Triggers Apoptosis in Transduced HL-1 Cells

**Figure 4:**
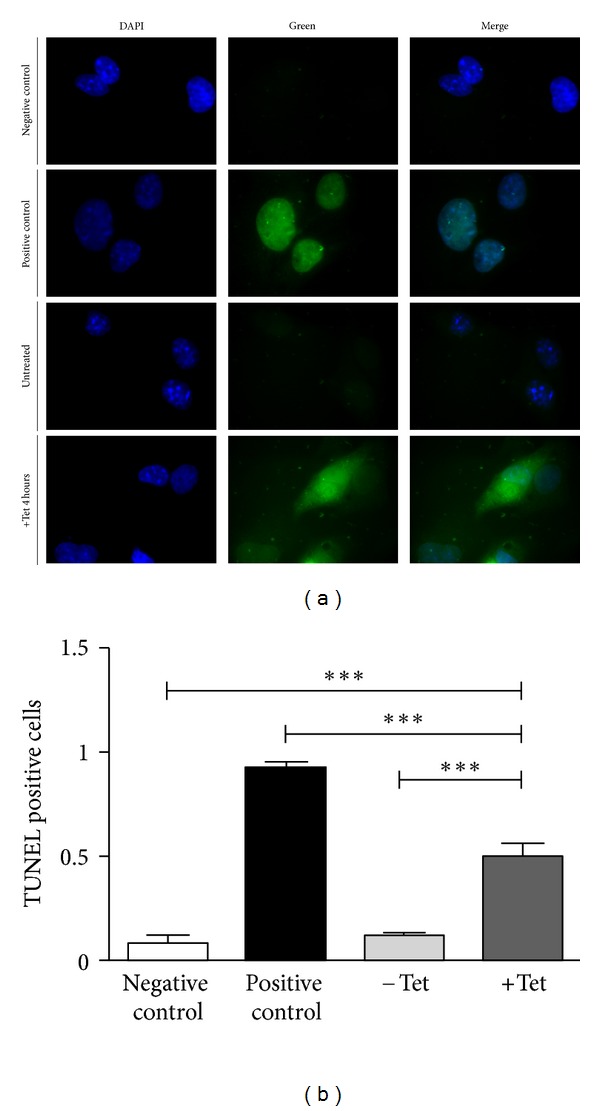
Measurement of apoptotic cell death. The amount of apoptotic cell death was determined after 4 hours exposure to 1 *μ*g/mL Tet by using the deoxynucleotidyl transferase-mediated dUTP nick end labeling (TUNEL), a standard method for evaluating DNA fragmentation. Following fluorescent labeling, cells were fixed on slides and analyzed by fluorescence microscopy. (a) Cells stained with DAPI (nuclear dye), fluorescein-labeled dUTP, and merging of the two channels. (b) Quantification of TUNEL-positive cells, expressed as the fraction of green fluorescent cells over nonfluorescent cells in each of the 6 fields counted for each condition. Negative control: nontransduced cells; positive control: cells treated with DNaseI; −Tet: HL-1 cells transduced with pNRTA vector and nontreated with Tet; +Tet: HL-1 cells transduced with pNRTA vector and treated with Tet for 4 hours.

To provide evidence that Tet induction of AIFsh expression was effectively leading to cell death by apoptosis, we performed a TUNEL, an immunofluorescence assays on transduced HL-1 cells (Figure 4(a)).

This analysis showed a strong fluorescent signal, corresponding to DNA fragmentation, 4 hours after Tet addition in approximately 50% cells (Figure 4(b)) (consistent with the decrease in cell survival found in the cell counts). In contrast, no signal was observed in either nontransduced or transduced but Tet-untreated cells. Statistical analysis showed that the difference in fluorescence was highly significant (*P* < 0.001) when induced cells were compared to noninduced cells or the negative control, while no significance was found when comparing the fluorescence of the negative control with that of uninduced cells.

These results showed that pTY2-TRE-AIFsh is an efficient and reliable *in vitro* system for inducing apoptosis of cardiomyocytes in a tightly regulated manner. The system consists of a lentiviral vector carrying a dual-promoter system that allows specific induction and restricted the expression of genes in cardiomyocytes. Induction was achieved by means of rtTA-regulated transcription of a cell type-specific promoter, whereas the transgene expression was driven by the TRE. In comparison to other gene delivery systems, in which the transfected genome is delivered in two separate plasmids, dual-promoter lentiviral vectors with modular design boast several advantages as they allow easy replacement of the transgenes and permit stable and regulatable expression of both transcriptional units in the recipient cells.

The evidence that neither latent expression from TRE in the absence of Tet (in HL-1 cells) nor the administration of Tet in transduced HeLa cells significantly altered the cells viability strengthens the validity of our system as a potential molecular tool for further *in vivo* studies.

Lentiviral vectors are well known as tools to deliver and stably express transgenes in a variety of cell types, including cells of cardiac lineage [14], and have been successfully used in a number of gene therapy studies showing their ability to sustain long-term transgene expression *in vivo *(reviewed in [23]). Moreover, the type of vector developed here is suitable for “lentivirus trangenesis,” an interesting strategy recently proposed as an alternative to direct microinjection of DNA into pronuclei [8]. Lentiviruses can efficiently transfer and integrate cDNAs into the host cell genome of oocytes and early embryos, thus making possible the genetic manipulation of animals other than mice, including large mammals. The transgene can then be activated or deactivated at any time of postnatal life simply by administering or withdrawing Tet. In agreement with this idea, Tet-regulated transgene activation has been successfully used in several transgenesis approaches *in vitro* and *in vivo*. These studies also demonstrated long-term responsiveness of the inducible system to Tet (reviewed in [24, 25]).

Wencker et al. have shown, in genetically modified mice, that very low levels of myocyte apoptosis are sufficient to cause a lethal, dilated cardiomyopathy in otherwise normal hearts [5], supporting the hypothesis that programmed cell death plays a major role in clinical heart failure [2, 3]. Therefore, a potential application of our construct would be the generation of clinically relevant, large animal models of dilated cardiomyopathy, which are currently unavailable and urgently required to study pathophysiological alterations and test new therapeutic interventions [26]. Alternatively, the dual-promoter system can be delivered to somatic cells during the adult life. In such a case, it might be necessary to produce an ad hoc vector to enhance the efficiency of the transduction of adult cardiomyocytes, which have proven almost completely refractory to conventional vector systems [27].

## 4. Conclusions

Our results offer a new and interesting tool that can be exploited to study apoptosis-related pathological conditions. Given the potential development of lentivirus transgenesis to develop novel and engineer existing animal models in the near future, the lentiviral construct described here may pave the way to produce clinically relevant models for further dilated cardiomyopathy research.
